# High precision in epileptic seizure self-reporting with an app diary

**DOI:** 10.1038/s41598-024-66932-y

**Published:** 2024-07-09

**Authors:** Nicolas Zabler, Lauren Swinnen, Andrea Biondi, Yulia Novitskaya, Elisa Schütz, Nino Epitashvili, Matthias Dümpelmann, Mark P. Richardson, Wim Van Paesschen, Andreas Schulze-Bonhage, Martin Hirsch

**Affiliations:** 1 Department of Neurosurgery, Epilepsy Center, Medical Center - University of Freiburg, Faculty of Medicine, University of Freiburg, Freiburg, Germany; 2 Department of Microsystems Engineering (IMTEK), Faculty of Engineering, University of Freiburg, Freiburg, Germany; 3 Laboratory for Epilepsy Research, KU Leuven, Leuven, Belgium; 4 Department of Basic and Clinical Neuroscience, Institute of Psychiatry, Psychology and Neuroscience, King’s College London, London, UK; 5 Department of Neurology, University Hospitals Leuven, Leuven, Belgium

**Keywords:** Epilepsy, Seizure diary, Electronic diary, Performance, Seizure counting, Overreporting, Neuroscience, Diseases of the nervous system, Epilepsy, Health care, Diagnostic markers, Data acquisition

## Abstract

People with epilepsy frequently under- or inaccurately report their seizures, which poses a challenge for evaluating their treatment. The introduction of epilepsy health apps provides a novel approach that could improve seizure documentation. This study assessed the documentation performance of an app-based seizure diary and a conventional paper seizure diary. At two tertiary epilepsy centers patients were asked to use one of two offered methods to report their seizures (paper or app diary) during their stay in the epilepsy monitoring unit. The performances of both methods were assessed based on the gold standard of video-EEG annotations. In total 89 adults (54 paper and 35 app users) with focal epilepsy were included in the analysis, of which 58 (33 paper and 25 app users) experienced at least one seizure and made at least one seizure diary entry. We observed a high precision of 85.7% for the app group, whereas the paper group’s precision was lower due to overreporting (66.9%). Sensitivity was similar for both methods. Our findings imply that performance of seizure self-reporting is patient-dependent but is more precise for patients who are willing to use digital apps. This may be relevant for treatment decisions and future clinical trial design.

## Introduction

One of the most prevalent neurological disorders globally is epilepsy, affecting nearly 1% of the world's population (7, 6 per 1000 people)^1,2^. Epilepsy is characterized by epileptic seizures, which can have a generalized or a focal onset in the brain, and manifest in a variety of forms. These seizures result from abnormal synchronous neuronal activity in the brain, though their pathological mechanisms are not yet fully understood. Individuals with epilepsy face an increased risk of injury and premature death, as well as comorbidities such as depression, anxiety, and cognitive impairments^3^.

While 60% of epilepsy cases can be effectively managed with long-term treatment using anti-seizure medications (ASMs) to suppress seizure activity^4^, the remaining cases require interventions such as resection of brain regions, neurostimulation, dietary changes, or alternative treatments to achieve a balance between reducing seizure frequency and managing treatment-related side effects.

Documentation of seizure frequency and clinical aspects is crucial for diagnosis and treatment. Seizure diaries, kept by people with epilepsy or their caregivers, have been the clinical standard to provide information about seizures experienced in everyday life. While one third of people with epilepsy do not document their seizures, two thirds of them do so to primarily monitor their condition^5^. It is also the usual method to determine seizure-related outcomes in clinical trials^6^.

Despite this significant role of seizure diaries, previous research has shown that about 50% of seizures are not documented^6,7^. This phenomenon of underreporting may be caused by seizure-induced lack of awareness, postictal inability to recall seizures, particularly during nighttime^8^, or by adverse drug effects^7^. Meanwhile, seizure overreporting is less commonly reported^6^, yet may additionally have a negative impact on the precision and validity of patient-based seizure documentation.

With the advent of digitalization, health apps have emerged as a modern approach to support people with neurological disease, especially people with epilepsy^9^. Numerous publications on epilepsy health apps report a variety of features and their potential impact on diagnosis, treatment, and patients’ well-being^9–14^. For example, medication reminders can improve adherence, while systematic data collection can contribute to a more consistent overview of seizure events^15^. As an alternative to paper-based seizure diaries, app-based seizure diaries have provided a novel approach of capturing seizure data which is also widely accepted by patients. Studies even reported more precise seizure documentation in terms of date and time compared to patients’ statements during their visit to the clinic and expected more reliable data^12,16^. However, there is no evidence whether the use of a digital seizure diary leads to improved documentation performance. Nonetheless, Karoly et al.^17^ have demonstrated that individual seizure risk can be forecasted using self-reported data collected from seizure apps, also highlighting additional use cases of digital diaries.

To the best of our knowledge there is no study that assesses the documentation performance of the two approaches—conventional paper-based diaries and modern app-based diaries—using video electroencephalography (video-EEG) as the gold standard. This exploratory study aims to provide insights in self-reporting seizures and to quantitatively identify documentation performance of patients using paper- or app-based seizure diaries.

## Material and methods

### Study design

This was a two-center retrospective study aiming to assess the performance of patients self-reporting their seizures using paper-based and app-based diaries. The data used in this study were obtained from participants of the in-hospital phase of the SeizeIT2 trial (clinicaltrials.gov NCT04284072) conducted in Leuven, Belgium, and Freiburg, Germany. The primary objective of SeizeIT2 was to evaluate a wearable, behind-the-ear seizure detection system (Sensor Dot; Byteflies, Belgium) in people with epilepsy during their stay in the epilepsy monitoring unit (EMU) by comparing it to gold standard video-EEG and seizure diaries^18^. Written informed consent was obtained from all patients or their legal guardians. The study was approved by the Ethics Committee Research UZ/KU Leuven (S63631) and the Ethics Committee at the University of Freiburg (279/20). All research was conducted in accordance with the Declaration of Helsinki.

### Data collection

During their stay in the EMUs of the tertiary epilepsy centers, participants had to choose between two methods to self-report their seizures: either a paper-based or an app-based seizure diary (Helpilepsy; Neuroventis, Belgium) installed on their smartphones. Both diaries were based on questionnaires in the form of free-text forms (see Supplementary Material Fig. S1). For the current study only the seizure diary related questions from both the paper and app-based diaries were used. Participants using the paper form were not reminded to report their seizures, while participants using the app diary received a push notification each day at 6 PM to complete this questionnaire. The paper questionnaires could be continuously edited by the participants during the day, conversely, once the app-based group had completed the questionnaire, they could no longer change their answers. Nevertheless, they could additionally self-report seizures through the app's standard seizure diary feature by entering details such as date, time, and seizure type, and use this information to fill out the questionnaire.

### Dataset creation

All diary entries were first manually assigned by L.S. and N.Z. to their corresponding electroclinical seizures (classified according to International League Against Epilepsy (ILAE) guidelines^19^) on the video-EEG, based on time information. For the assignment every reasonable temporal difference between the diary entry and its corresponding seizure timestamp was accepted. Diary entries that were correctly assigned to their seizures were considered true positives (TPs). Diary entries that could not be matched to a video-EEG documented seizure were considered false positives (FPs). Seizures documented with video-EEG, for which no diary entry was available were considered false negatives (FNs). All diary entries that were not relevant to seizures in the specific temporal context (e.g. “I am not feeling well today”) were ignored. For every participant, information about age, sex, prior diary experience, anti-seizure medication (ASM), duration of their stay in the EMU, and the number of their seizures and diary entries were extracted. Further, for every seizure, details such as seizure type, duration, originating hemisphere, involved lobes, and preictal awake/asleep state were extracted.

Participants were eligible for inclusion if they were adults with a diagnosed form of focal epilepsy, who filled out an app-based or paper-based seizure diary by themselves while participating in the SeizeIT2 study in Leuven or Freiburg and their diary was available. Additional inclusion criteria were having at least one seizure during their stay in the EMU while participating in the study OR/AND having made at least one (seizure-relevant) diary entry. Excluded were participants with generalized epilepsy due to very low documentation sensitivity^18^, participants supported by caregivers due to expected oversensitivity and children as they were under parental supervision.

### Data analysis

#### Assessment of performance metrics of seizure diaries

The performances of participants of both groups were assessed using metrics based on the concept of standards for testing and clinical validation of seizure detection devices^20^, namely:

1. *Sensitivity* TP/(TP + FN), indicates how many seizures were correctly reported in the diary
2. *Precision* TP/(TP + FP), indicates how many of the reported events were in fact seizures
3. *F1-Score* 2TP/(2TP + FP + FN), harmonic mean of sensitivity and precision
4. *False alarm rate per 24 h (FAR24)* Number of FPs during the visit in the EMU

At the level of seizures, the metrics were calculated by including all values independently, regardless of the participants. Further, at the level of participants, metrics were determined on a participant-specific basis, and subsequently, the arithmetic mean and standard deviation were computed across all participants. Participant-level metrics are therefore independent of the total number of seizures and diary entries of a participant. Sensitivity, requiring at least one seizure occurrence, and precision, requiring at least one diary entry, were calculated only if a participant fulfilled both criteria. F1-score and FAR24 were also calculated for participants who met only one of these criteria.

#### Statistical analysis of potential influencing parameters

The statistical association between meta parameters and the performance metrics were investigated to find possible influencing factors, for the overall cohort, and for the specific groups. Differences in proportions of meta parameters between the app and the paper group were analyzed.

At the level of seizures, the preictal awake/asleep state of the participant, level of awareness (according to the seizure classification by the ILAE), seizure duration, hemispheric lateralization, and lobar origin were investigated. Since this information was only present for seizures (TPs, FNs) and not for reported non-seizures (FPs), all the analyzed associations at seizure level only reflect their influence on sensitivity, not on precision. At the level of participants, age, sex, diary experience, ASM, and the seizure frequency were investigated for potential effects on sensitivity and precision of seizure documentation.

Nonparametric statistical testing was applied (Mann–Whitney U test, Spearman rank correlation, Chi-squared test [χ^2^]) and significance levels were adjusted for multiple comparisons using the Bonferroni correction.

## Results

### Dataset

**Figure 1:**
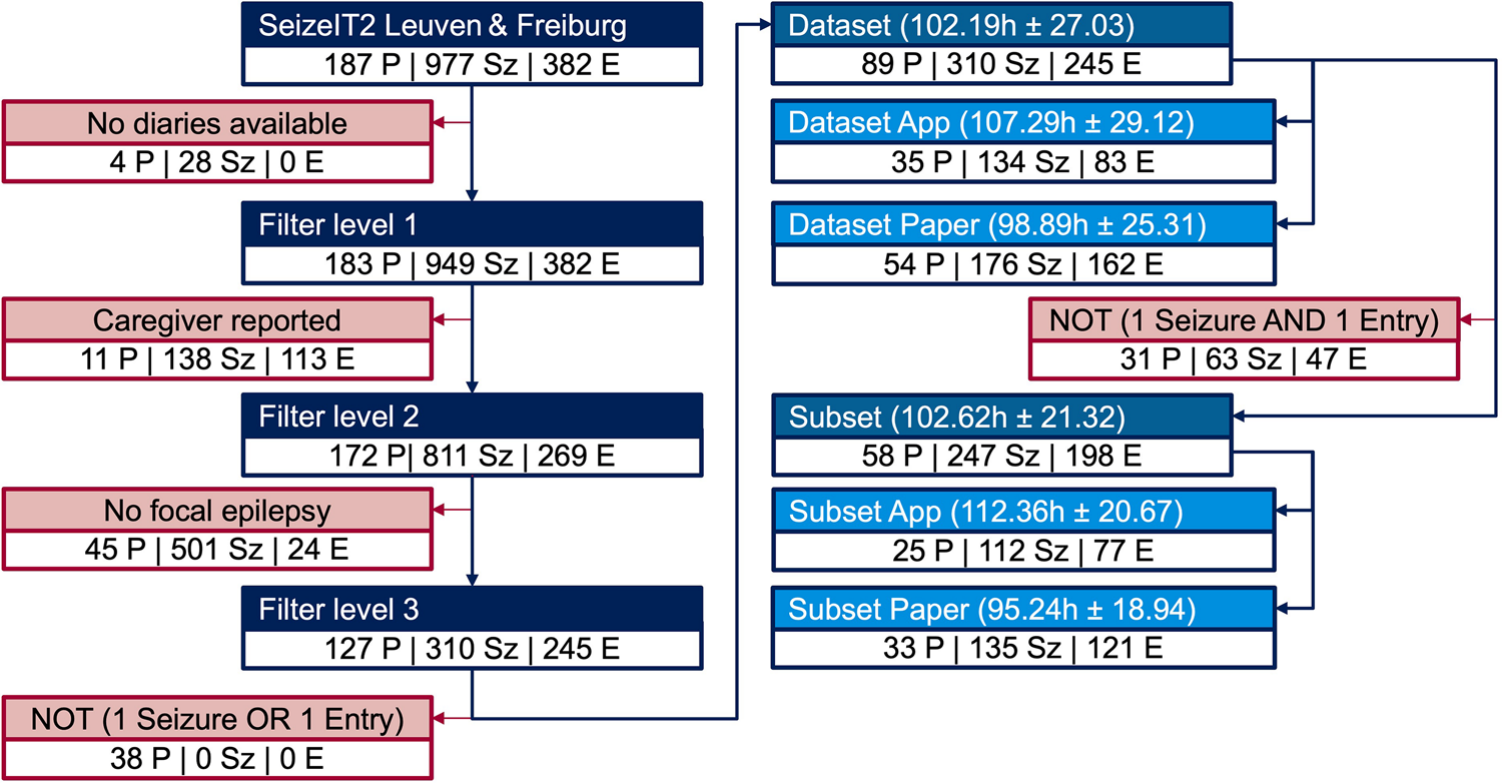
Dataset creation. This diagram shows the consecutive steps performed to filter the data for the analysis. After excluding (in red) participants (P) without diaries or those with diaries filled out by caregivers, the final two datasets comprised individuals with focal epilepsy who self-reported their seizures (Sz) through diary entries (E) either in the app or on paper. These individuals had at least one seizure (not seizure free) OR made at least one diary entry (active use of the diary), i.e., the dataset, or had at least one seizure AND made at least one diary entry, i.e., the subset. The average duration (in hours) and standard deviation of the participants' stay in the EMU can be found in brackets of the two sets and their subgroups.

Figure 1 illustrates the consecutive filtering steps leading to the final dataset, and a subset that meet the necessary criteria for calculating the metrics. The dataset, including 89 participants with at least one seizure OR at least one diary entry made, comprises a total of 310 seizures and 245 diary entries. The subset, including 58 participants with at least one seizure AND at least one diary entry made, contains 247 seizures and 198 diary entries.

### Performance metrics

**Figure 2:**
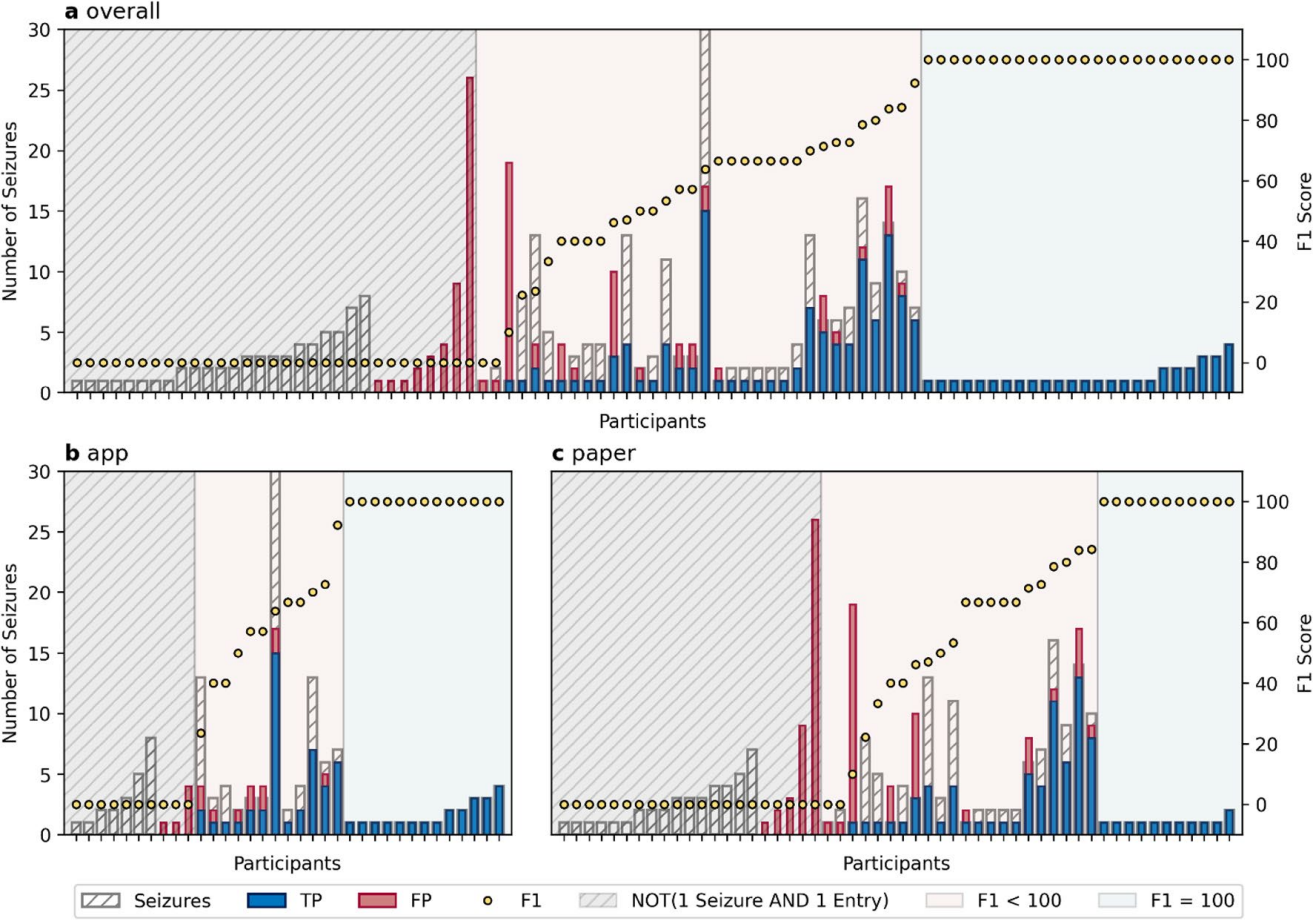
Individual performances. The plots reveal the seizure number, TPs and FPs as well as the F1-score for each participant sorted by an increasing F1-score per participant (from left to right). The top panel (**a**) shows all seizure diaries in total, while the bottom panel is separated per diary form (**b** app, **c** paper). The gray-striped area highlights the participants who were excluded from further analysis (filtered out from dataset to subset). The green area highlights the perfect documenters (F1-score = 100%) and the red area highlights the non-perfect documenters (F1-score < 100%).

The numbers of True Positives (TPs), False Positives (FPs), and False Negatives (FNs) are shown in Table 1, and the resulting performance metrics are presented in Table 2. For the complete dataset, sensitivities were below 50%, independent of the method, while for the app group, precision at seizure level was high (79.5%). Within the subset at seizure level the app group had an overall higher precision (85.7% vs. 66.9%), higher F1-score (69.8% vs. 63.3%), and lower FAR24 (0.09 vs. 0.31) compared to the paper group. Sensitivities were very similar (app group: 58.9%, paper group: 60.0%). At participant level, the same phenomena were observed within the subset, however, the app group reported with a higher average sensitivity of 76.5% compared to the paper group (69.9%). Moreover, individuals showed a high precision for both methods (app: 88.7%, paper: 82.7%), whereas we observed a low variation between the individuals in the app group (± 19.9%) and high variation (± 32.2%) in the paper group. Figure 2 reveals that 25.8% (23/89) of all participants in the dataset had unreported seizures only, while 9.0% (8/89) of the participants reported only seizures without video-EEG correlate. These 34.8% (31/89, marked in gray) of the participants had to be excluded, due to the inability to calculate sensitivity and precision. The remaining 65.2% of the participants (58/89) comprising the subset, experienced at least one seizure (i.e. were not seizure free) and made at least one diary entry (i.e. used the diary actively). These 58 participants were categorized into perfect documenters (F1-score equals 100%, 24/58, marked in green) and non-perfect documenters (F1-score < 100%, 34/58, marked in red). No patient in the group of the perfect documenters had more than 5 seizures, while the group of non-perfect documenters had a highly variable number of seizures ranging from 1 up to 30. Of the 34 non-perfect documenters, 26 were underreporting, i.e., participants which were reporting less than their actual seizure count. Of these 26 underreporting non-perfect documenters, 17 reported no FPs, 7 participants reported FPs and TPs, and two reported only FPs. The remaining 8 participants among the non-perfect documenters were overreporters, i.e., participants reporting more seizures than their actual number of seizures. Classified into the app and paper group (subset), underreporters who reported only FPs were solely observed in the paper group (n = 2, 6%). In the app group, there were 5 (20%) underreporters reporting FPs and TPs, while there were two (6%) in the paper group. Furthermore, there were two (8%) overreporting non-perfect documenters in the app group, while there were 6 (18%) in the paper group being responsible for 76.6% (36/47) of its group FPs.

**Table 1:** Matching matrix showing the TPs, FPs, and FNs for the overall group, both diary forms, and both filter levels.

Video-EEG	Overall		App		Paper	
	Positive	Negative	Positive	Negative	Positive	Negative
Dataset 1 Seizure OR 1 Entry						
Positive	147	163	66	68	81	95
Negative	98	–	17	–	81	–
Subset 1 Seizure AND 1 Entry						
Positive	147	100	66	46	81	54
Negative	51	–	11	–	40	–

**Table 2:** Performance metrics calculated for seizure level and participant level on both filter levels.

Metric	Seizure level			Participant level mean (± s.d.)		
	Overall	App	Paper	Overall	App	Paper
Dataset 1 Seizure OR 1 Entry						
Sensitivity	47.42	49.25	46.02	–	–	–
Precision	60.00	79.52	50.00	–	–	–
F1-Score	52.97	60.83	47.93	47.71 (± 41.84)	58.82 (± 40.86)	41.60 (± 40.93)
FAR24	0.26	0.11	0.36	0.25 (± 0.73)	0.12 (± 0.23)	0.32 (± 0.91)
Subset 1 Seizure AND 1 Entry						
Sensitivity	59.51	58.93	60.00	72.76 (± 31.48)	76.53 (± 27.66)	69.90 (± 33.81)
Precision	74.24	85.71	66.94	85.31 (± 27.75)	88.73 (± 19.86)	82.72 (± 32.24)
F1-Score	66.07	69.84	63.28	73.21 (± 28.61)	80.00 (± 24.05)	68.07 (± 30.65)
FAR24	0.21	0.09	0.31	0.20 (± 0.60)	0.10 (± 0.18)	0.28 (± 0.77)

### Influencing factors

All statistical tests were applied on the subset (see Supplementary Material Tables S7-10).

#### Seizure level

We observed a significantly lower number of seizures documented in the seizure diaries if they happened while the participants were asleep compared to when they were awake (44.3% [47/106] vs. 70.9% [100/141], ${\chi }_{1}^{2}= 16.66$, *p* < 0.001). This association was present in both groups, but was significant only in the app group (72.9% [54/74] vs. 49.6% [12/38], ${\chi }_{1}^{2}= 16.11$, *p* < 0.001), and not in the paper group (68.7% [46/67] vs. 51.5% [35/68], ${\chi }_{1}^{2}= 3.47$, *p* = 0.063). The level of awareness during seizures was not associated with the number of reported seizures. Note that 50 seizures were excluded from this analysis because the level of awareness could not be determined according to the guidelines. Furthermore, we found no association of the number of reported seizures with their origin, lateralization or duration.

Between the groups, the preictal awake/asleep state, the seizure origin, and the seizure duration did not differ statistically. However there were fewer seizures with preserved awareness in the app group than in the paper group (21.1% [20/95] vs. 47.1% [48/102], ${\chi }_{1}^{2}= 13.59$, *p* < 0.001) and there were more seizures originating from the left hemisphere (76.4% [55/72] vs. 41.8% [38/91], ${\chi }_{1}^{2}= 18.27$, *p* < 0.001) in the app group than in the paper group.

#### Participant level

Seizure frequency had a significant negative correlation with sensitivity (Spearman rank correlation, $r=-0.63$, *p* < 0.001, n = 58) but not with precision (Spearman rank correlation,$r=-0.17$, *p* = 0.212, n = 58). The significant negative correlation of seizure frequency with sensitivity was evident in both groups (app: [Spearman rank correlation, $r=-0.60$, *p* < 0.001, n = 25], paper: [Spearman rank correlation, $r=-0.62$, *p* < 0.001, n = 33]). No significant associations were found between sensitivity or precision with the participants’ age, sex, diary experience or ASM, neither in the overall cohort, nor in the groups. Further none of these parameters did differ between the groups.

## Discussion

We found that seizures were reported with high precision by individuals with focal epilepsy who used an app-based seizure diary. Seizures reported in paper diaries were associated with a low precision, as some individuals tended to overreport their seizure activity. In addition, the study revealed that people with epilepsy with a higher seizure frequency showed a lower documentation sensitivity. Our study also corroborates previous research which found moderate sensitivity (approximately 50%) of seizure diaries and an effect of preictal awake/asleep state on sensitivity.

### App-based diaries associated with high self-reporting precision

Sensitivity at seizure level was around 60% for both methods which aligns with prior studies^7^. However, individuals of the app group showed higher sensitivity, despite the fact that these patients more frequently had focal unaware seizures and seizures originating from the left hemisphere (both parameters which have been reported to be associated with a low self-reporting performance). Importantly, our study demonstrated that reported seizures are associated with a higher precision (85.7%) for the app-based diary and a lower precision when paper was used (66.9%). This finding aligns with studies on diaries for headaches and pain, which showed significantly fewer errors in digital forms compared to paper forms^21,22^.

While at participant level individuals showed a high precision for both methods, the higher variation in the paper group stems from the non-perfect documenters with a tendency to overreport their actual seizure count (6 participants). These findings suggest that patients who are willing to use an app for seizure documentation, it may offer a more reliable option. Furthermore, FAR24 indicated only one false reported seizure every two weeks on average, highlighting the app’s usefulness for clinical trials. Statistical analysis did not reveal differences between the groups at participant level, i.e. in age, sex, prior diary experience, seizure frequency, or medication.

### Impact of overreporting in seizure self-reporting

There were six patients in the paper group who had considerably overreported their seizure frequencies meaning a low self-reporting precision and were responsible for 76% of their groups total FP count. Therefore, understanding the mechanisms of how and why non-seizures (FPs) are reported, is crucial to comprehend self-reporting precision. Prior research has predominantly focused on sensitivity of seizure diaries, whereas the number of FPs and precision has been barely explored in relation to overreporting.

Multiple studies noted a significant disparity between clinical and documented events in adult patients with focal epilepsy, concluding that most patients underestimate their seizures^23–25^. However, Pizarro et al.^26^ and Elmali et al.^27^, both focused on generalized seizures and reported that 57% and 37.5% of their cohorts, respectively, were overreporting. Lacking objective measures, the authors hypothesized that over-attentiveness due to patients’ or caregivers’ insecurity in missing potential seizures in the EMU might be responsible. Identifying factors contributing to FPs in our study, independent of the method, was challenging due to the absence of meta parameters for non-seizure events: participants might have had limited knowledge of seizure semiology, perceiving trivial feelings and movements as seizures^28^. From our data, it indeed appears that uncertain subjective experiences might have served as potential indicators for seizures. Further, hospitalization could have induced hypervigilance and over-sensitivity^26,27^.

In contrast, in an outpatient setting, Cook et al.^25^ found overreporting as part of the reason for a lack of correlation of self-reported and objectively documented seizures as assessed by mobile intracranial EEG. Recently, Hannon et al.^29^ observed seizure overreporting in a large outpatient cohort. Specifically in patients with diagnosed focal epilepsy 43% of the reported events were FPs, which aligns with our findings (40% FPs in the overall cohort). These two studies strongly suggest that overreporting is not an artifact limited to the conditions of inpatient monitoring.

Patient education regarding seizure semiology, coupled with a neurologist's careful explanation of typical clinical signs, could significantly enhance self-reporting precision. In fact, in our cohort, one reported FP was classified as a psychological non-epileptic seizure (PNES). More extensive description of events in the diary might allow to distinguish PNES from epileptic seizures based on certain features. Further, this observation also elucidates the fact that certain epileptic seizures, characterized by a clear epileptic clinical pattern, can lack an electrographic correlate in surface EEG signals, particularly evident in focal seizures^30^. Finally, the diary form may have influenced the participants' tendency to overreport, especially in our study setting with a short inpatient stay. Reporting seizure activity with a hastily written paper note can leave significant room for errors. Conversely, utilizing a new app with its comprehensive range of functions and the associated mental effort, may have encouraged participants to focus more on accurate self-reporting, thereby reducing the margin for error.

### Sensitivity is influenced by awake/asleep state and seizure frequency

We observed an overall self-reporting sensitivity of 59.5%. Poor documentation sensitivity, indicating underreporting, is primarily attributed to seizure-induced lack of awareness and nocturnal seizures^7^. Schulze-Bonhage et al.^8^ recently confirmed circadian effects of underreporting seizures, aligning with our findings. Furthermore, Kerling et al.^31^ had already shown that seizures during sleep are significantly less reported compared to awake seizures. Unlike Hoppe et al.^23^, our study did not find a significant association between the level of awareness and the number of reported seizures. This may be due to the variations in the definitions of awareness in the guidelines used^28^, and to the limited number of seizures with information on the state of awareness. Furthermore, multiple studies revealed a negative effect on documentation sensitivity from seizures originating from the left temporal lobe^31–33^, which was not found in the seizures analyzed for this study. Interestingly, although the app group was associated with more seizures originating from the left hemisphere and impaired awareness—both factors linked to underreporting—a lower sensitivity was not found. This possibly indicates an overall positive effect of the app on documentation sensitivity.

At the participant level, we found a negative correlation between seizure frequency and sensitivity. Higher seizure frequency leading to lower sensitivity may be attributed to a ‘diary fatigue effect’^34^. This effect becomes evident when comparing seizure counts between perfect documenters and non-perfect documenters. It further implies a careful interpretation of reports from patients with high seizure counts when valid documentation is critical, e.g. the outcome of a patient’s treatment or the definition of inclusion criteria for clinical trials.

### Pro and cons of seizure diaries: future directions

Study outcomes suggest that self-reporting seizures using an app may be associated with a high precision, particularly for patients who are willing to use digital apps. Although generally people with epilepsy show interest in documenting seizures with apps^35^, the actual utilization is still low^36^. Explaining the benefits of long-term use and providing training^37,38^ and education^39,40^ on these tools, might increase utilization (see Supplementary Material). Our data may thus support the recommendation of apps for seizure documentation in clinical practice and particularly in clinical trials. Besides our findings, app-based diaries show several other benefits. These are, among others, better readability, increase in temporal accuracy due to predefined time stamps^12^, a lower risk of losing data, and enhanced accessibility for health care providers^14^. Further, systematically collected data would allow for personalized, data-driven therapy with the help of automatic analysis tools. This technology might be useful for clinical and research purposes, serving as a patient-reported outcome and aiding in the evaluation of medication. Specifically in therapeutic trials, research has already shown that high app-based diary compliance can be achieved^41^.

Nevertheless, the use of seizure diaries to self-report seizures lacks sensitivity^7^, as recently also shown for ultra-long periods in the outpatient setting^42^. Further, the performance depends on the individual patient and even with a high diary compliance, self-reporting is still affected by seizure unawareness. One way to overcome this challenge is through the utilization of wearable seizure detection devices that can objectively measure seizure signs based on biosignals such as electrocardiogram, acceleration, or low-channel EEG^9^. Looking ahead, an optimal solution may involve the integration of app-based diaries into wearable seizure detection systems^7^. This combined approach has the potential to enhance sensitivity and minimize the number of false positives (FPs), ultimately leading to an overall improved performance in seizure documentation. Moreover, objective seizure detection based on biosignals can be complemented with patients’ descriptions of precipitating factors to their seizures and even possible videos of the event taken by a caregiver, harnessing the full potential of these methods.

### Limitations

Given the retrospective nature of this study, it inherently entails certain limitations. First, participants were not randomly assigned to one of the two diaries; instead, they were allowed to self-select their preferred diary. As a result, the study allowed the identification of associations but precluded the establishment of causal inferences. Whereas letting patients choose the documentation method may induce a selection bias, this reflects clinical practice and the self-reporting performance in either diary is not independent of the patient’s preferences. Second, app diary results were based on questionnaire responses in the app, potentially affecting performance (see Methods). Besides, the daily reminders provided only to app users might have influenced results. However, Hoppe et al.^23^ reported that such daily reminders do not necessarily have an influence on documentation performance, leaving it undecided whether this factor contributed to superior precision of the app group. Third, the controlled lab conditions of EMUs and changes in ASM dosages differ from real-life home environments and might have potentially influenced seizure reporting. Finally, generalizability is limited as only focal epilepsies were analyzed and as patients in whom caregivers supported seizure documentation were excluded from this study. Further research in larger datasets may also address possible effects of seizure clustering on their documentation, which was not included here.

## Conclusion

In conclusion, app-based seizure diaries have the potential to enhance patients' performance in self-reporting seizures, particularly in terms of precision. Accurate seizure diaries allow neurologists to make better decisions in daily clinical care. Increased utilization of epilepsy health apps will expand databases, contributing to further validation of app-based approaches in assessing their relevance for treatment decisions and clinical trials.

### Supplementary Information

Supplementary Information.

## Data Availability

The data that support the findings of this study are not publicly available due to data sharing agreements with patients included in this study. However, data will be made available in a de-identified format upon reasonable request to the corresponding author.
