# Supplementary material for: High precision in epileptic seizure self-reporting with an app diary
Source: Sci Rep. 2024 Jul 9;14:15823. doi: 10.1038/s41598-024-66932-y (PMC11233562; doi:10.1038/s41598-024-66932-y)
Supplement: Supplementary file 1 — Supplementary Information. [file 41598_2024_66932_MOESM1_ESM.pdf]

Supplementary Information

High Precision in Epileptic Seizure Self-Reporting with an App Diary

Nicolas Zabler, Lauren Swinnen, Andrea Biondi, Yulia Novitskaya, Elisa Schütz, Nino Epitashvili, Matthias Dümpelmann, Mark P. Richardson, Wim Van Paesschen, Andreas Schulze-Bonhage, Martin Hirsch

SeizeIT2 Trial

The original data for this work was obtained from the multicenter trial SeizeIT2 (clinicaltrials.gov NCT04284072). The primary goal of SeizeIT2 was to clinically validate a biopotential and motion recording wearable device (Byteflies Sensor Dot) for detection of epileptic seizures in the epilepsy monitoring unit (EMU) and at home. The participation criteria for the study were:

Inclusion Criteria

- Subjects (4+ years old) with refractory epilepsy who are admitted to the hospital for clinically indicated long-term video-EEG assessment or presurgical evaluation, and a high likelihood of experiencing seizures during the EMU Phase
- For subjects continuing into the Home Phase: successful recording of their habitual seizures with Sensor Dot during the EMU Phase
- For subjects continuing into the Home Phase: the ability to keep an e-diary

Exclusion Criteria

- Known allergies to any of the biopotential electrodes or adhesives used as part of the study protocol
- Having an implanted device, such as (but not limited to) a pacemaker, cardioverter defibrillator (ICD), and/or neural stimulation device because Sensor Dot contains magnets that could interfere with the operation of these devices
- Women who are pregnant

For the retrospective analysis presented in this work, SeizeIT2 data from the centers in Leuven and Freiburg, collected during the EMU phase, were utilized, as these centers were the only ones to employ both app and paper seizure diaries during the trial

Figure S1. Dataset creation using data from SeizeIT2 trial.

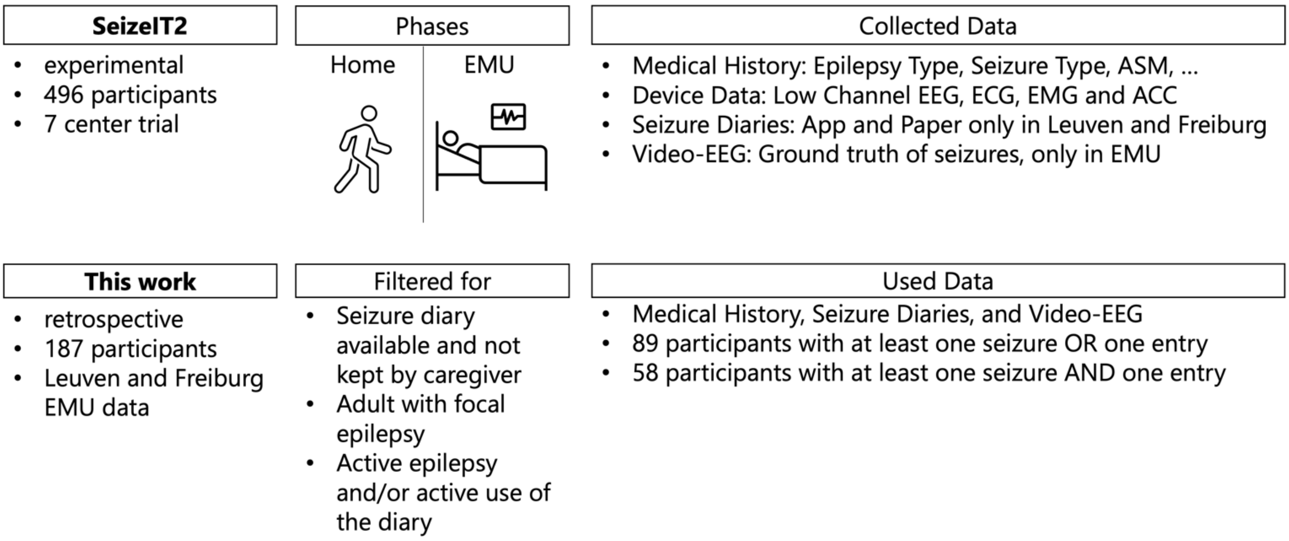

Daily Questionnaire

Figure S2. Seizure Diaries. Daily study questionnaires in SeizeIT2 represented as a paper seizure diary (A) and an app seizure diary (B) in Leuven. On the left, the original version in Dutch (the paper version was digitally modified for anonymity reasons). On the right, translated in English.

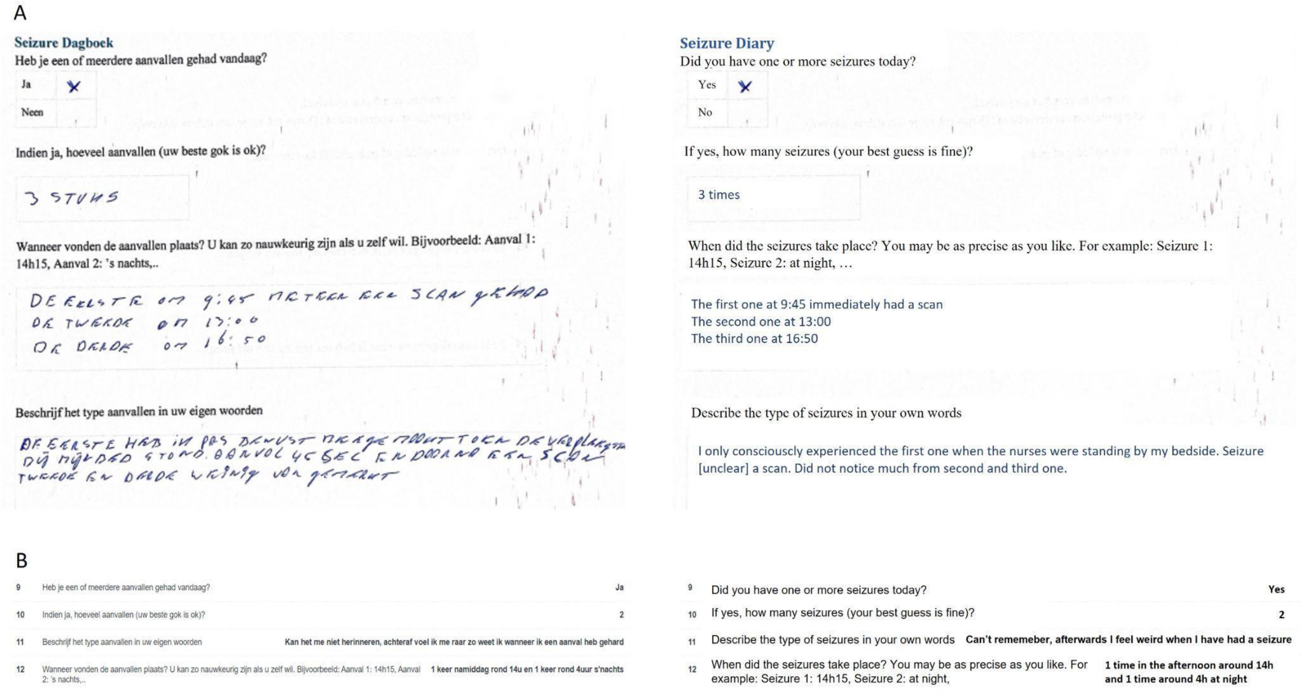

Metrics

Table S1. Mathematical Definitions of Performance Metrics. The table shows the definitions of the performance metrics on seizure level and on participant level. TP: true positive, FP: false positive, FN: false negative, N: number of participants and FAR24: false alarm rate per 24 hours. Note that 'precision' is used here as in the context of machine learning and information retrieval and should not be mistaken for temporal precision. It can be understood interchangeably as 'positive predictive value', more commonly used in the medical field.

	**Seizure level**	**Participant level**
**Sensitivity**	$\frac{TP}{TP+FN}$	$\frac{1}{N}\sum_{i=1}^{N}\frac{TP_i}{TP_i+FN_i}$
**Precision (Positive Predictive Value)**	$\frac{TP}{TP+FP}$	$\frac{1}{N}\sum_{i=1}^{N}\frac{TP_i}{TP_i+FP_i}$
**F1-Score**	$\frac{2TP}{2TP+FP+FN}$	$\frac{1}{N}\sum_{i=1}^{N}\frac{2TP_i}{2TP_i+FP_i+FN_i}$
**FAR24**	$\frac{FP}{Duration\ in\ EMU}\cdot 24h$	$\frac{24h}{N}\sum_{i=1}^{N}\frac{FP_i}{Duration_i\ in\ EMU}$

Meta Parameter

Table S2. Meta Parameter for dataset creation and data analysis.

Seizure Level Meta Parameters	Value Range
Report	Timestamp, precision: varying between min to h
Seizure	Timestamp, precision: s
Awake/Asleep State	awake; asleep; unknown
Level of awareness	aware; impaired; unknown
Hemisphere	left; right; bilateral; unknown
Lobe	frontal; temporal; parietal; occipital; mixed; unknown
Duration	EEG onset to offset; precision: s
**Participant Level Meta Parameters**	**Value Range**
Age	precision: years
Sex	male; female; unknown
EMU duration	precision: h
Seizures	Amount of seizures
Entries	Amount of diary entries
Seizure Frequency	Amount of seizures/EMU duration; precision: 1/d
TPs	Amount of TPs
FPs	Amount of FPs
FNs	Amount of FNs
Diary Experience	yes; no; unknown
ASM	levetiracetam; brivaracetam; other
**Filter Meta Parameters**	**Value Range**
Seizure onset	focal; generalized; unknown
Reporting person	person with epilepsy; caregiver; both
Diary form	app; paper; Not available

Datasets

Table S3. Dataset at seizure level.

Meta Parameters	Values		
	All	App	Paper
Seizures	310	134	176
Entries	241	79	162
TPs	147	66	81
FPs	98	17	81
FNs	163	68	95
Preictal Awake/Asleep State			
awake	179	82	97
asleep	131	52	79
unknown	0	0	0
Awareness			
aware	80	21	59
impaired	158	83	75
unknown	72	30	42
Hemisphere			
left	132	70	62
right	91	21	70
bilateral	14	4	10
unknown	73	39	34
Lobe			
frontal	65	37	28
occipital	6	6	0
parietal	1	1	0
temporal	168	65	103
mixed	52	20	32
unknown	18	5	13
Seizure Duration [s]	74.14 (SD 69.76)	75.89 (SD 70.46)	73.56 (SD 69.42)

Table S4. Dataset at participant level.

Meta Parameters	Values		
	All	App	Paper
Participants	89	35	54
Age	35.79 (SD 13.84)	35.46 (SD 10.97)	39.30 (SD 15.32)

Sex			
male	42	14	28
female	347	21	26
other	0	0	0
EMU duration [h]	102.19 (SD 27.03)	107.29 (SD 29.12)	98.89 (SD 25.31)
Seizures	3.48 (SD 4.51)	3.83 (SD 5.52)	3.26 (SD 3.75)
Entries	2.75 (SD 4.51)	2.37 (SD 3.13)	3.00 (SD 5.23)
TPs	1.65 (SD 2.72)	1.89 (SD 2.83)	1.50 (SD 2.66)
FPs	1.10 (SD 3.55)	0.49 (SD 0.92)	1.50 (SD 4.48)
FNs	1.83 (SD 2.69)	1.94 (SD 3.36)	1.76 (SD 2.17)
Seizure Frequency [1/h]	0.0400 (SD 0.0529)	0.0406 (SD 0.0599)	0.0399 (SD 0.0477)
Diary Experience			
yes	33	15	18
no	56	20	36
ASM			
LEV or BRV	61	21	40
Other	28	14	14

Table S5. Subset at seizure level.

Meta Parameters	Values		
	All	App	Paper
Seizures	247	112	135
Entries	194	73	121
TPs	147	66	81
FPs	51	11	40
FNs	100	46	54
Preictal Awake/Asleep State			
awake	141	74	67
asleep	106	38	68
unknown	0	0	0
Awareness			
aware	68	20	48
impaired	129	75	54
unknown	50	17	33
Hemisphere			

left	93	55	38
right	70	17	53
bilateral	12	2	10
unknown	72	38	34
Lobe			
frontal	61	36	25
occipital	6	6	0
parietal	1	1	0
temporal	126	56	70
mixed	35	8	27
unknown	18	5	13
Seizure Duration [s]	74.15 (SD 75.67)	79.38 (SD 75.56)	69.81 (SD 75.78)

Table S6. Subset at participant level.

Meta Parameters	Values		
	All	App	Paper
Participants	58	25	33
Age	35.81 (SD 11.97)	34.60 (SD 9.71)	36.73 (SD 13.52)
Sex			
male	28	10	18
female	30	15	15
other	0	0	0
EMU duration [h]	102.62 (SD 21.32)	112.36 (SD 20.67)	95.24 (SD 18.94)
Seizures	4.26 (SD 5.23)	4.48 (SD 6.25)	4.01 (SD 4.39)
Entries	3.41 (SD 4.19)	3.08 (SD 3.38)	3.66 (SD 4.75)
TPs	2.53 (SD 3.02)	2.64 (SD 3.04)	2.46 (SD 3.05)
FPs	0.88 (SD 2.60)	0.44 (SD 0.77)	1.22 (SD 3.37)
FNs	1.72 (SD 2.97)	1.84 (SD 3.67)	1.64 (SD 2.36)
Seizure Frequency [1/h]	0.0446 (SD 0.0540)	0.0435 (SD 0.0616)	0.0454 (SD 0.0474)
Diary Experience			
yes	23	12	11
no	35	13	22
ASM			
LEV or BRV	41	15	26
Other	17	10	7

Statistical Results

For the statistical analysis, age, seizure frequency, seizure duration, as well as sensitivity and precision, were considered as continuous variables. Awake/asleep state (awake, asleep), awareness (aware, impaired), hemisphere (left, right), lobe (temporal, frontal), sex (m, f), diary experience (yes, no), ASM (LEV or BRV, other), as well as TP/FN (1, 0), were treated as dichotomously distributed variables with the corresponding binary categories in parentheses. To avoid Type I errors, the conservative Bonferroni method was applied to the four test families in terms of the concept of family-wise error rate. Note that seizures were removed from statistical analysis if the variable was determined as unknown (hemisphere, lobe, awareness). Additionally, categories with group sizes considered too small for statistical analysis were excluded (seizures with bilateral onset, occurring in the occipital or parietal lobes).

Table S7. Statistical associations within the subset at seizure level. *Significance was reached when the p-value was lower than the Bonferroni-adjusted alpha of 0.05/15.

Test	Group	Variables	N	P-value	Statistic
Chi-squared	all	TP/FN, Awake/Asleep State	247	0.0000 *	16.6596
Chi-squared	app	TP/FN, Awake/Asleep State	112	0.0001 *	16.1057
Chi-squared	paper	TP/FN, Awake/Asleep State	135	0.0626	3.4681
Chi-squared	all	TP/FN, Awareness	197	1.0000	0
Chi-squared	app	TP/FN, Awareness	95	1.0000	0
Chi-squared	paper	TP/FN, Awareness	102	0.9900	0.0002
Chi-squared	all	TP/FN, Hemisphere	163	0.3335	0.9353
Chi-squared	app	TP/FN, Hemisphere	72	0.1459	2.1143
Chi-squared	paper	TP/FN, Hemisphere	91	1.0000	0
Mann-Whitney U Test	all	TP/FN, Seizure Duration	247	0.4553	7762
Mann-Whitney U Test	app	TP/FN, Seizure Duration	112	0.3006	1342.5
Mann-Whitney U Test	paper	TP/FN, Seizure Duration	135	0.0845	2571.5
Chi-squared	all	TP/FN, Lobe	187	0.1993	1.6475
Chi-squared	app	TP/FN, Lobe	92	0.9477	0.0043
Chi-squared	paper	TP/FN, Lobe	95	0.0267	4.9101

Table S8. Statistical differences between groups within the subset at seizure level. *Significance was reached when the p-value was lower than the Bonferroni-adjusted alpha of 0.05/5.

Test	Group	Variables	N	P-value	Statistic
Chi-squared	all	Awake/Asleep State, Diary Form	247	0.0135	6.1005
Chi-squared	all	Awareness, Diary Form	197	0.0002*	13.5898
Chi-squared	all	Hemisphere, Diary Form	163	0.0000*	18.2864
Mann-Whitney U Test	all	Seizure Duration, Diary Form	247	0.1228	8423
Chi-squared	all	Lobe, Diary Form	187	0.0868	2.9332

Table S9. Statistical associations within subset at participant level. *Significance was reached when the p-value was lower than the Bonferroni-adjusted alpha of 0.05/30.

Test	Group	Variables	N	P-value	Statistic
Spearman Correlation	all	Sensitivity, Age	58	0.9378	-0.0105
Spearman Correlation	all	Precision, Age	58	0.5811	-0.074
Spearman Correlation	app	Sensitivity, Age	25	0.8587	0.0375
Spearman Correlation	app	Precision, Age	25	0.2186	-0.255
Spearman Correlation	paper	Sensitivity, Age	33	0.9075	-0.021
Spearman Correlation	paper	Precision, Age	33	0.8628	0.0313
Mann-Whitney U Test	all	Sensitivity, Sex	58	0.8491	408
Mann-Whitney U Test	all	Precision, Sex	58	0.0959	506.5
Mann-Whitney U Test	app	Sensitivity, Sex	25	0.3221	58
Mann-Whitney U Test	app	Precision, Sex	25	0.9438	76.5
Mann-Whitney U Test	paper	Sensitivity, Sex	33	0.6483	147.5
Mann-Whitney U Test	paper	Precision, Sex	33	0.0532	179
Spearman Correlation	all	Sensitivity, Seizure Frequency	58	0.0000*	-0.6306
Spearman Correlation	all	Precision, Seizure Frequency	58	0.2121	-0.1663
Spearman Correlation	app	Sensitivity, Seizure Frequency	25	0.0014*	-0.6024
Spearman Correlation	app	Precision, Seizure Frequency	25	0.0846	-0.3518
Spearman Correlation	paper	Sensitivity, Seizure Frequency	33	0.0001*	-0.6233
Spearman Correlation	paper	Precision, Seizure Frequency	33	0.9941	0.0013
Mann-Whitney U Test	all	Sensitivity, Diary Experience	58	0.8925	411
Mann-Whitney U Test	all	Precision, Diary Experience	58	0.4463	363.5
Mann-Whitney U Test	app	Sensitivity, Diary Experience	25	0.1261	51.5
Mann-Whitney U Test	app	Precision, Diary Experience	25	0.6043	70
Mann-Whitney U Test	paper	Sensitivity, Diary Experience	33	0.1852	154.5
Mann-Whitney U Test	paper	Precision, Diary Experience	33	0.6220	110
Mann-Whitney U Test	all	Sensitivity, ASM	58	0.9060	355.5
Mann-Whitney U Test	all	Precision, ASM	58	0.5168	317.5
Mann-Whitney U Test	app	Sensitivity, ASM	25	0.6745	82.5
Mann-Whitney U Test	app	Precision, ASM	25	0.7246	80.5
Mann-Whitney U Test	paper	Sensitivity, ASM	33	0.9446	89
Mann-Whitney U Test	paper	Precision, ASM	33	0.2671	70

Table S10. Statistical differences between groups within the subset at participant level. *Significance was reached when the p-value was lower than the Bonferroni-adjusted alpha of 0.05/5.

Test	Group	Variables	N	P-value	Statistic
Mann-Whitney U Test	all	Age, Diary Form	58	0.8627	401
Chi-squared	all	Sex, Diary Form	58	0.4051	0.6931
Mann-Whitney U Test	all	Seizure Frequency, Diary Form	58	0.6043	379
Chi-squared	all	Diary Experience, Diary Form	58	0.3899	0.7392
Chi-squared	all	ASM, Diary Form	58	0.4591	0.548

Digital Tools Survey

Introduction

The goal of this two-center retrospective study was not only to compare the performance of paper-based and app-based seizure diaries, but also to understand patients' views on epilepsy health apps. Therefore, at the end of the study, participants who chose the app-based seizure diary were also asked to provide feedback about the app by completing a digital questionnaire.

Methods

Participants' experience with the app were assessed using a digital questionnaire (see Table below) delivered via the app at the end of the study. Participants were asked to complete it and they were also allowed to skip specific questions if they were not sure about their answers. The questionnaire included 8 questions focusing on 4 main topics: Interaction [Q1], ease to use (Q3, Q4), understanding and support (Q5, Q6, Q7), and future use (Q2, Q8). Finally, participants had the possibility to complete a free text box to report suggestions to improve the app [Q9]. Descriptive analysis was performed on the collected data (mean, median, standard deviation and percentage were reported) while main suggestions were summarized for Q9.

Results

Of the 35 participants using the app (dataset), 25 decided to fill out the questionnaire about their experience with the app (average age of 35y; sex 14F, 8M). There was no questionnaire for the paper group (54 participants). Participants rated the app as easy to use (Q4) and to install (Q3) and, on average, they used it at least once a day (Q1). Most of them would recommend the app to other people with epilepsy (Q2), and 7 (31.8%, out of 22) would be happy to continue using it after the study ends at home with only one (4.5%, out of 22) who would not (Q8). Participants reported low levels of perceived assistance while using the app (Q6), believed the app improved only a bit their knowledge about epilepsy (Q5), and did not feel much better prepared for their future discussion with their neurologists (Q7). Furthermore, one participant proposed including a function to report triggers, two participants suggested it would be important to receive training on how to use the app at the start, and three participants reported minor technical difficulties related with the readability or writing information via the app (Q9).

Discussion

Although low utilization of the app may stem from its perceived lack of simplicity compared to paper, users described the app as easy to use, experiencing only minor technical or practical issues. This aligns with Macea et al. (2023), who reported a 60% adherence to using the Helpilepsy® app daily for several months among 15 people with epilepsy, confirming the app's acceptance. In line with recent studies (Bruno et al., 2018; Simblett et al., 2019; Egenasi et al., 2023), one of our participants suggested that apps can help reduce common issues with paper diaries, such as forgetting to complete them. However, our results showed that participants did not feel assisted, their knowledge about epilepsy did not improve, and they did not feel more prepared for future interactions with clinicians. This may be due to the brief usage of the app during their hospital stay (mean of 4.56 days) and the app's lack of direct feedback, data summaries, or educational tools during this period. Conversely, factors such as education (Le Marne et al., 2018; Yoo et al., 2020) and training (Khan et al., 2020; Dedeken et al., 2021) are crucial to improve acceptability and to increase long-term usage.

Conclusion

People with epilepsy widely accept epilepsy health apps and recognize the benefits of documenting their seizures using app-based diaries compared to paper diaries. However, the revelation of greater potential in terms of assistance, knowledge, and shared decision-making requires long-term use and education. Consequently, medical personnel have to provide training on these tools to lower the burden of usage and increase utilization.

Table S11. Digital Tools Survey Summary. From left to right: the question, original scale, adapted scale for scoring, number of participants who replied, and score obtained. All scores were reported as average, standard deviation and median apart for Q8 where we reported the percentage.

	Questions	Original Scale	Adapted Scale	N	Mean	SD	Median
Q1	Approximately how many times a day do you use the Helpilepsy application (including to answer the daily questionnaires)?	Not once<br>Once<br>2-5 times<br>> 5 times a day	0<br>1<br>2<br>3	19	1.2	0.4	1
Q2	How likely are you to recommend the application to other people with epilepsy?	Extremely Likely<br>Neutral<br>Extremely Likely	1<br>5<br>10	25	6.9	1.6	6

**Q3**	How easy was it to install the application?	very difficult<br>difficult<br>neutral<br>easy<br>very easy	1<br>2<br>3<br>4<br>5	24	4.2	0.6	4
**Q4**	How easy was it to use the application?	very difficult<br>difficult<br>neutral<br>easy<br>very easy	1<br>2<br>3<br>4<br>5	23	3.7	0.8	4
**Q5**	Did you have the feeling the application helped you to better understand your disease?	not at all<br>only a little<br>to some extent<br>rather much<br>very much	1<br>2<br>3<br>4<br>5	21	2.0	1.0	2
**Q6**	Did you feel better assisted/supported when you regularly used the application?	not at all<br>only a little<br>to some extent<br>rather much<br>very much	1<br>2<br>3<br>4<br>5	21	2.3	0.9	2
**Q7**	Did you have the feeling the application helped you to have better discussions with your doctor or medical caregivers?	not at all<br>only a little<br>to some extent<br>rather much<br>very much	1<br>2<br>3<br>4<br>5	22	2.0	1.0	2
	**Questions**	**Original Scale**	**Adapted Scale**	**N**	**Yes (%)**	**Maybe (%)**	**No (%)**
**Q8**	Will you continue to use the application even after completing the study?	yes<br>maybe<br>no	2<br>1<br>0	22	7<br>(31.8)	14<br>(63.6)	1<br>(4.5)

References

(1) Macea, J.; Bhagubai, M.; Broux, V.; De Vos, M.; Van Paesschen, W. In-hospital and Home-based Long-term Monitoring of Focal Epilepsy with a Wearable Electroencephalographic Device: Diagnostic Yield and User Experience. Epilepsia 2023, 64 (4), 937–950. https://doi.org/10.1111/epi.17517.

(2) Dedeken, P.; Muhumuza, S. N.; Sebera, F.; Umwiringirwa, J.; Bitunguhari, L.; Tierens, H.; Teuwen, D. E.; Boon, P. A. J. M. Long-Term Impact of Single Epilepsy Training on Knowledge, Attitude and Practices: Comparison of Trained and Untrained Rwandan Community Health Workers. Int J Public Health 2021, 66, 645598. https://doi.org/10.3389/ijph.2021.645598.

(3) Yoo, S.; Lim, K.; Baek, H.; Jang, S.-K.; Hwang, G.; Kim, H.; Hwang, H. Developing a Mobile Epilepsy Management Application Integrated with an Electronic Health Record for Effective Seizure Management. International Journal of Medical Informatics 2020, 134, 104051. https://doi.org/10.1016/j.ijmedinf.2019.104051.

(4) Khan, A.; Peechatka, A.; Dias, N.; Lima, V.; Seddo, M.; Inja, A.; Dallabrida, S. M. Patient Perspective in the Development of Electronic Patient-Reported Outcomes (ePROs) in Seizure Disorders: A Patient-Centric Approach. PPA 2020, Volume 14, 13–21. https://doi.org/10.2147/PPA.S222642.

(5) Simblett, S. K.; Bruno, E.; Siddi, S.; Matcham, F.; Giuliano, L.; López, J. H.; Biondi, A.; Curtis, H.; Ferrão, J.; Polhemus, A.; Zappia, M.; Callen, A.; Gamble, P.; Wykes, T. Patient Perspectives on the Acceptability of mHealth Technology for Remote Measurement and Management of Epilepsy: A Qualitative Analysis. Epilepsy & Behavior 2019, 97, 123–129. https://doi.org/10.1016/j.yebeh.2019.05.035.

(6) Le Marne, F. A.; Butler, S.; Beavis, E.; Gill, D.; Bye, A. M. E. EpApp: Development and Evaluation of a Smartphone/Tablet App for Adolescents with Epilepsy. Journal of Clinical Neuroscience 2018, 50, 214–220. https://doi.org/10.1016/j.jocn.2018.01.065.

(7) Bruno, E.; Simblett, S.; Lang, A.; Biondi, A.; Odoi, C.; Schulze-Bonhage, A.; Wykes, T.; Richardson, M. P. Wearable Technology in Epilepsy: The Views of Patients, Caregivers, and Healthcare Professionals. Epilepsy & Behavior 2018, 85, 141–149. https://doi.org/10.1016/j.yebeh.2018.05.044.

(8) Egenasi, C. K.; Moodley, A. A.; Steinberg, W. J.; Joubert, G.; Egenasi, C. The Perceptions and Attitudes of Patients with Epilepsy to the Use of a Seizure Diary, South Africa. Open Access. https://doi.org/10.4102/safp.v65i1.5503.
